# Socioeconomic and demographic predictors of resident knowledge, attitude, and practice regarding arthropod-borne viruses in Panama

**DOI:** 10.1186/s12889-018-6172-4

**Published:** 2018-11-14

**Authors:** A. Whiteman, A. Mejia, I. Hernandez, J. R. Loaiza

**Affiliations:** 1 Department of Geography & Earth Sciences, University of North Carolina at Charlotte, 9201 University City Blvd, Charlotte, NC 28223 USA; 2 Smithsonian Tropical Research Institute, P.O. Box 0843-03092, Balboa, Ancón Republic of Panama; 3 Instituto de Investigaciones Científicas y Servicios de Alta Tecnología (INDICASAT AIP), P.O. Box 0843-01103, Panamá, República de Panamá; 4 Programa Centroamericano de Maestría en Entomología, Universidad de Panamá, Panama City, República de Panamá

**Keywords:** Socioeconomic factors, Demographic characteristics, Arthropod-borne viruses, Knowledge, Attitude, And practice, Panama City

## Abstract

**Background:**

We sought to identify if socioeconomic and demographic factors play a role in resident knowledge, attitude, and practice regarding Dengue, Chikungunya, and Zika in order to inform effective management procedures for disease prevention in Panama, a middle-income tropical country in Central America. All three are arthropod-borne viruses transmitted by *Aedes* mosquito vectors present in the focal region of Panama City, the largest city in Central America and an urban region of extreme socioeconomic polarization.

**Methods:**

Between November 2017 and February 2018, we administered standardized, anonymous knowledge, attitude, and practice surveys to 263 residents split between two neighborhoods of high socioeconomic status (SES) and two neighborhoods of low SES. We then summed the knowledge, attitude, and practice scores respectively, and used linear and logistic regressions to quantify relationships with socioeconomic and demographic factors.

**Results:**

Low-SES neighborhoods with high proportions of low income residents, residents over 70 years old had lower mean knowledge scores compared to other groups. Furthermore, residents in neighborhoods of low SES reported more mosquito biting relative to residents in neighborhoods of high SES, yet comparably lower level of concerns for disease transmission. Additionally, knowledge was lower for the more novel emergent threats of Chikungunya and Zika, compared to the endemic Dengue.

**Conclusion:**

Findings suggest that low-SES neighborhoods with high proportions of low income, low education, and elderly residents should be targeted for outreach programs designed to prevent DENV, CHIKV, or ZIKV in Panama City. These outcomes support our initial hypotheses as lower relative knowledge and fewer practices related to the prevention of Dengue, Chikungunya, and Zika were found in low-SES neighborhoods. There is also a widespread lack of adequate knowledge regarding these diseases as well as low levels of concern in areas of highly reported mosquito biting. We provide suggestions for taking neighborhood socioeconomic status and specific aspects resident health literacy and attitude into account for creating more effective outreach campaigns as both endemic and novel arthropod-borne disease rates continue to increase throughout Latin America.

**Electronic supplementary material:**

The online version of this article (10.1186/s12889-018-6172-4) contains supplementary material, which is available to authorized users.

## Background

Arthropod-borne viruses (e.g., Arboviruses) are responsible for over 1 million deaths a year globally, in addition to causing hundreds of billions of dollars in societal costs [1]. Dengue virus (DENV), Chikungunya virus (CHIKV), and Zika virus (ZIKV) are three particularly significant arbovirus threats. They are primarily transmitted to humans, the principal host, by *Aedes aegypti* and *Aedes albopictus* mosquitoes, whose ranges and capacity to spread disease have greatly expanded in recent decades as a result of globalization [2], urbanization [3], and climate change [4]. The risk of disease outbreak due to these arboviruses is not only reliant on the presence of infected mosquitoes, but also requires a susceptible host population to sustain transmission. For comprehensive risk assessments of DENV, CHIKV and ZIKV, *Aedes* vector surveillance can be supplemented with information on socioeconomic and demographic characteristics of human communities, as these are likely key predictors of viral transmission dynamics [5, 6]. Observing the distribution of medical knowledge, fear of transmission, and disease prevention practices across communities of varying socioeconomic and demographic characteristics can inform management procedures to areas where public education and outreach may be more effective means of disease prevention than vector control.

Knowledge, Attitude, and Practice (KAP) surveys have been used for decades to gauge community risk for numerous medical issues ranging from HIV/AIDS [7] to tobacco use [8]. For arbovirus transmission risk assessments, KAP surveys can be used to estimate knowledge of vector behavior and disease characteristics, attitudes or fears towards the vectors and viruses, and applications of any methods they use to prevent themselves from encountering mosquito vectors. Survey responses can be linked with data on the respondent’s personal attributes (e.g. age, sex, education level, financial situation, medical history) to identify common trends or differences among groups in an attempt to define risk predictors [9]. KAP surveys have been commonly applied in malaria zones in Asia and Sub-Saharan Africa, identifying education and income as a direct socioeconomic predictors of knowledge of malaria transmission as well as quantifying the relationships between past exposure to the disease and future preparedness [10–13]. Demographic characteristics such as age have also been found to be linked with KAP, specifically revealing that knowledge is lowest among older respondents [14]. KAP studies in endemic Dengue Fever regions have shown that a community can be knowledgeable of risk, yet not take any precautions to avoid Dengue transmission [15, 16]. Further studies have found the reverse, where communities can have very little knowledge of Dengue transmission, yet employ high levels of preventatives measures [17]. This seeming contradiction indicates the importance of conducting assessments of community KAP at each individual region of interest, rather than extrapolating results from other studies. One key gap in the literature is the understanding of variations in resident KAP as it pertains to multiple spatially coexisting diseases. As both vectors and pathogens continue to arise in novel locations, it is important to understand how residents view emerging diseases compared to endemic ones. To date, yet few KAP studies on the emergent threats of ZIKV and CHIKV have taken place in the Americas, where dengue is considered endemic [18, 19].

In this study, we attempt to estimate how resident knowledge, attitudes, and practices concerning three arboviruses vary between socioeconomically differing communities. Understanding a community’s capacity to withstand disease in the context of socioeconomics is an important aspect of social determinants of health [20]. Economic stability [21, 22], education [23], social context [24], and the built environment [25] all contribute to the relative health risk of an individual. They impact one’s access to healthcare [26], social support [27], public safety [28], and even exposure to pathogens [29, 30]. Therefore, examining the increasing risk of arbovirus transmission under the lens of social determinants of health can provide meaningful feedback for public health authorities in regions where residents may be at a greater risk due to specific socioeconomic conditions. Here we address this subject in Panama City, Panama, a region where both arbovirus transmission and socioeconomic polarization are particularly high.

While DENV has been endemic in Panama since 1970, CHIKV was first reported in 2014, followed by ZIKV in 2015. All three arboviruses are now present in Panama City, the largest city in Central America and a hub of international trade and tourism, with 2.5 million people arriving to the city from abroad in 2017. Additionally, Latin America has the highest income inequality of any region on Earth [31, 32], with Panama having the second most unequally distributed wealth in the region, with a Gini coefficient of 0.50. Panama City specifically has a considerable division between high and low income communities, ranking in the top 20 of cities on Earth with the most unequally distributed wealth. About 48% of the country lives below the poverty line while the wealthiest 20% own 50% of the nation’s overall wealth [32, 33]. This has led to vastly different neighborhood environments and community demographics, including highly wealthy and educated high-rise communities situated in close proximity to communities of extreme poverty. With a constant influx of potential hosts, stark socioeconomic contrasts, a climate supporting year-round mosquito development, and risk of three separate arboviruses, Panama City represents an ideal location to apply KAP surveys as a mean of assessing outbreak preparedness in the region. We frame our study within the context of social determinants of arbovirus transmission risk by administering KAP surveys to contrasting neighborhoods within the same urban region. Based on the results of previous investigations, we expect lower relative knowledge and fewer practices related to the prevention of DENV, CHIKV, and ZIKV among residents of communities of lower SES. We also expect relative knowledge about emergent CHIKV and ZIKV to be lower than that of endemic DENV.

## Methods

### Study areas and sampling design

In order to isolate social determinants of health as drivers of KAP, we identified four neighborhoods in Panama City by creating a socioeconomic index of all counties (e.g., Corregimientos) out of two key metrics that have been used previously to describe local socioeconomic conditions for health disparity research [34, 35]: 1) average household income and 2) percentage of residents with bachelor degrees or higher. We obtained the values of these variables for each “Corregimiento” from the National Institute of Statistics and Census (http://www.contraloria.gob.pa/inec/, 2010). We then normalized and averaged them across all Corregimientos in order to attain a percentile ranking of SES for the Metropolitan Panama City. We then selected four focal neighborhoods, two in the 95th percentile and two in the 5th percentile based on similarity in population density and normalized difference vegetation index. The unselected neighborhoods in each stratum (*n* = 15–20) consisted of study populations that were too small or too far from the city center to be appropriate for the study. The two high SES neighborhoods, Costa Del Este and Punta Pacifica, primarily consist of high-rise apartment buildings, office buildings, and gated housing communities interspersed by parks and vacant lots. The two low SES neighborhoods, Altos De Las Torres and Boca La Caja, primarily consist of conjoined single-story family homes and businesses (Table 1). Built-up land or impervious surface is the primary land type across all four neighborhoods. Costa Del Este, Punta Pacifica, and Boca La Caja are located along the coast, while Altos De Las Torres is located 3.8 km inland. The average monthly family income in the high SES neighborhoods is over $1400, while the average monthly family income in the low SES neighborhoods is less than $350. Similarly, in the high SES neighborhoods, 34% of residents have a bachelor degree or higher, while 3.5% of residents in the low SES neighborhoods have a bachelor degree or higher.

**Table 1:** Characteristics of each focal neighborhood

	Costa Del Este (high SES)	Punta Pacifica (high SES)	Altos De Las Torres (low SES)	Boca La Caja (low SES)
Land Use Type	Single homes	High rises	Single homes	Slum/informal settlements
Total Population	8699	3961	8063	2475
Population Density (people/km^2^)	2361	6531	6399	6513
Housing Density (houses/km^2^)	639	1927	1501	1713
Percent with Bachelors Degree	36%	32%	1.3%	6.4%
Employment rate	66%	53%	62%	60%
NDVI	−0.013	−0.020	−0.019	− 0.021

A 100 m grid was created in each selected neighborhood, with each vertex serving as a focal point for surveys. The first person to answer the door at the four closest properties were approached for surveys and if rejected, the adjacent property was approached. In multi-family structures, such a high-rise apartment, we recruited by approaching the first four people that exited the building entrance upon our arrival, with permission from building managers. This allowed for a more complete spatial coverage of each neighborhood as well as a more representative cross section of the community than would have been obtained if certain demographic proportions had been targeted. However, while we did not target specific proportions of groups, this may be interpreted as an example of quota sampling, which is itself a form of non-probability sampling. Non-probability sampling is a commonly used tool in public health [36–39], and while advantages have been identified [40], it is an often criticized tool based on its inability to allow for extrapolation or generalization of results [41, 42]. While these shortcomings are acknowledged, this study was intended to provide direct assistance to local public health authorities, who benefited from results based on true population proportions. Further studies on this topic using competing methods, such as probability sampling, are invited. Based on the layout of the grid and number of vertices, we attempted to complete 60 surveys (15 grid points) in Boca la Caja, 72 (18 grid points) in Altos de las Torres, 72 (18 grid points) in Costa del Este, and 64 (16 grid points) in Punta Pacifica.

### Instrument

The questionnaire was developed based on reviewing previous KAP studies, incorporating a combination of subject matter and question formats that have been successfully applied in Africa, Asia, Europe, and Latin America regarding DENV, ZIKV, and malaria risk [10, 12, 15, 17, 43–45]. Additionally, it is designed to be a pilot for a wider scale survey on infectious disease risk across Panama to be conducted in the future. The questionnaire first involved gathering information on the resident’s demographic, educational, occupational and economic situation. Then, multiple-choice prompts measured the resident’s knowledge of DENV, CHIKV, and ZIKV. This involved asking if they were familiar with these diseases as well as whether or not they are preventable with a vaccine, curable with treatment, what their primary symptoms are, and how they are transmitted. Next, multiple choice questions assessed the resident’s knowledge of mosquito ecology, including the time of day they are most active. We then used Likert scales to gauge the resident’s worry of contracting each disease and whether they believe each disease should be a concern for the community. The next portion of the questionnaire asked the resident whether they implement any vector control strategy or mechanisms on their property, which specific mechanisms they use, and how often they are applied, and whether they think they are effective or not at limiting disease. This is not assessing their knowledge of the literature, but instead gauges their perception as to the effectiveness of vector control. The final questions asked where they have received most of their information on preventative tips, what they would do if they thought they had contracted one of the aforementioned diseases, and how many times they think they are bitten by a mosquito each day.

Informed written consent was obtained from each participant before the survey administered by a trained interviewer. The surveys were filled out in-person using pencil and paper between 9:00 am - 4:00 pm. While this may have incurred bias against individuals with full-time employment, there were safety risks that prohibited interviewers from surveying outside those hours.

### Data management and analyses

Survey answers were entered into Microsoft Excel once in full with an additional 50% entered alongside the original as validation. For the knowledge questions, correct answers include knowing there is no vaccine or therapeutic cure for any of the three diseases. At the time of the survey, a DENV vaccine was briefly provided with considerable publicity in parts of Brazil, but this was never offered in Panama; we expected our respondents to answer with regard to what was available to them. Correct answers for primary modes transmission are only mosquito for all three diseases. ZIKV does have other modes of potential transmission, yet vector-borne transmission is responsible for 95% of the basic reproductive number [46]. Correct answers for the primary symptoms of DENV were headache, fever, rash, muscle pain, or joint pain. Correct answers for the primary symptoms of CHIKV were headache, fever, and joint pain. Correct answers for the primary symptoms of ZIKV were fever, rash, joint pain, and conjunctivitis. Incorrect answers were provided for each question as well (see Additional file 1: Survey). Correct answers for the primary time of day for mosquito activity is anytime except night. The answers to the symptom questions were weighted so as not to disproportionately affect the results. Specifically, while correct answers to the other questions in the knowledge section were given a point value of one, each correct symptom was given a 0.25 point value so that all four correct symptoms summed to one, for each disease. The attitude questions were entered as they were answered using the Likert scales, scaled 1–7 (low to high). Respondents were asked to rank how worried they were about contracting each of the diseases, how much of a major problem they thought that contracting the diseases might be for their health, and how likely they were to seek medical attention if they believed they had contracted one of these diseases. The practice questions were entered similarly to the knowledge questions. Eight options were provided: frequently change the water in flower pots/vases, sleep under bed net at night, remove containers that accumulate clean water, eliminate tanks and puddles that accumulate water, drink from tightly closed water containers, keep windows and doors closed in the house, request fumigation, and other (a space for options not listed). A weighted value was recorded for each option they reported doing, similar to the knowledge section, where reporting participation in all preventative measures equaled a value of one. For the question of how often they apply each method, options were daily, weekly, every 2 weeks, monthly, and yearly. For the question of where they learned about preventative measures, they were given the option of TV, radio, newspaper, at work, in school, neighborhood campaign, family/friend, medical professional, or other. For the question of what they would do if they thought they had contracted one of the diseases, options were provided as: Centro de Salud (primary health center), private hospital, public hospital, or other. Finally, the number of times they report being bitten by a mosquito each day was recorded.

For data analyses, the limitations of quota sampling must be acknowledged. Because samples are not randomized, there are certainly concerns over equal variance. Studies that employ non-probability sampling widely apply the same significance testing procedures as those that use probability sampling, yet there are important considerations to be aware of [47]. Thus, we conduct our significance testing with the understanding that assumptions of equal variance cannot necessarily be met, due to the inherent design of the sampling, and we recommend readers interpret the results with the same precautions. Still, quota sampling has undeniable advantages [40, 48], specifically in its low cost and direct value to local public health authorities who prefer studies with representative samples of the communities they monitor.

We first used chi-square tests [49] to compare demographic and socioeconomic attributes between the two SES neighborhood categories (e.g. high, low). Then, the knowledge responses were summed to attain an overall knowledge score (0–18), which was used as the main dependent variable in the knowledge analyses, while the socioeconomic variables and demographic characteristics (Table 1; individual-scale), and the SES of the neighborhood (SES-scale) were used as predictors. We also assessed collinearity using variable inflation factors, and we report the models that contain solely significant and non-confounding relationships. Effects between overall knowledge score and socioeconomic and demographic predictors were assessed using generalized linear models (GLM; [50]) regressions. GLMs do not assume normality nor homogeneity of variance, and so would be appropriate for use in non-probability survey sample datasets. The attitude variables were assessed by summing and the Likert scale responses. Using the same socioeconomic variables, demographic characteristics, and neighborhoods as predictors, generalized linear models were then generated for the overall score and individual question scores. For the practice variables, generalized linear models with either logistics or Poisson linkages were used, depending on the format of the particular variable. Missing data was rare and thus was not imputed. All analyses were conducted in Stata IC version 15 [51].

## Results

### Socio-demographic characteristics of sample population

A total of 263 surveys were applied to residents of four focal neighborhoods of Panama City, between November 2017 and February 2018. Table 2 indicates the socioeconomic and demographic characteristics of the respondents, divided by neighborhood, respectively. Because the neighborhoods were chosen specifically for their socioeconomic differences and surveys were indiscriminately conducted based on location in the neighborhood rather than respondent attributes, there is significant variation between groups for most of demographic characteristics. While there was no significant difference in sex ratio overall, the high SES group had significantly more male respondents than the low SES group (*P* < 0.01). The ages of the respondents varied by SES, with the low SES group having higher proportions of older respondents compared to the high SES group (*P <* 0.01). Ethnicity varied in that there were higher proportions of white and indigenous respondents in the high SES group than the low SES group (*P* < 0.01). Respondents in the high SES group had completed more schooling than the respondents in the low SES neighborhoods (*P* < 0.01), yet both personal and family monthly income did not vary by SES group. This may be a result of inaccuracies in self-reported income [52].

**Table 2:** Socio-demographic characteristics of the focal neighborhoods

	Overall (%)	Boca La Caja (%)	Altos De Las Torres (%)	Punta Pacifica (%)	Costa Del Este (%)	Low ses average	High ses average	*P (SES-Level)*
N	263	59 (23)	72 (27)	69 (26)	63 (24)	65.5	66	
Head of the family	128 (49)	38 (29)	36 (28)	29 (23)	25 (20)	37	27	0.06
Sex								0.02
Male	148 (56)	33 (22)	28 (19)	47 (32)	40 (27)	36	43.5	
Female	115 (44)	26 (23)	44 (38)	22 (19)	23 (20)	35	22.5	
Age Bracket								0.00
18–35	100 (38)	8 (8)	35 (35)	28 (28)	29 (29)	21.5	28.5	
36–55	94 (36)	14 (15)	21 (22)	32 (34)	27 (29)	17.5	29.5	
56–70	39 (15)	17 (47)	9 (23)	6 (15)	6 (15)	13	6	
70+	30 (11)	20 (67)	7 (23)	3 (10)	0 (0)	12.5	1.5	
Number of People in Household								0.75
1	20 (8)	4 (20)	2 (10)	4 (20)	10 (50)	3	7	
2	42 (16)	10 (24)	9 (22)	12 (28)	11 (26)	9.5	11.5	
3	52 (20)	14 (27)	11 (21)	13 (25)	14 (27)	12.5	13.5	
4	53 (20)	9 (17)	18 (34)	18 (34)	8 (15)	13.5	13	
5	38 (15)	5 (13)	15 (39)	11 (29)	7 (19)	10	9	
6	22 (8)	10 (45)	6 (27)	4 (18)	2 (10)	8	3	
7	13 (5)	3 (23)	4 (31)	2 (16)	4 (30)	3.5	3	
7+	23 (9)	4 (18)	7 (30)	5 (22)	7 (30)	5.5	6	
Ethnicity								0.00
White	40 (15)	4 (10)	8 (20)	15 (37)	13 (33)	6	14	
African-Caribbean	27 (10)	2 (8)	11 (40)	10 (37)	4 (15)	6.5	7	
African-colonial	13 (5)	1 (8)	2 (15)	6 (46)	4 (31)	1.5	5	
Mestizo	147 (66)	49 (33)	45 (31)	24 (16)	29 (20)	47	26.5	
Indigenous	28 (11)	1 (4)	3 (11)	15 (53)	9 (32)	2	12	
Chinese/asian	3 (1)	1 (33)	1 (33)	1 (34)	0 (0)	1	0.5	
Other	2 (1)	0 (0)	0 (0)	0 (0)	2 (100)	0	1	
Did not disclose	3 (1)	0 (0)	2 (67)	0 (0)	1 (33)	1	0.5	
Marital status								0.79
Single	77 (29)	15 (25)	18 (25)	19 (26)	25 (40)	16.5	22	
Married	85 (33)	18 (31)	21 (30)	26 (37)	20 (32)	19.5	23	
Divorced	3 (1)	1 (2)	0 (0)	1 (1)	1 (2)	0.5	1	
Separated	4 (2)	1 (2)	0 (0)	1 (1)	2 (3)	0.5	1.5	
Widowed	12 (5)	5 (42)	1 (8)	1 (8)	5 (42)	3	3	
Free Union	80 (30)	13 (16)	30 (38)	20 (25)	17 (21)	21.5	18.5	
Highest Education Completed								0.03
None	11 (4)	9 (82)	1 (9)	0 (0)	1 (9)	5	0.5	
Some Primary School	2 (0)	1 (50)	0 (0)	0 (0)	1 (50)	0.5	0.5	
Finished Primary School	14 (5)	7 (50)	5 (35)	0 (0)	2 (15)	6	1	
Some High School	23 (9)	11 (48)	4 (17)	8 (35)	0 (0)	7.5	4	
Finished High School	69 (27)	18 (26)	29 (42)	12 (17)	10 (15)	23.5	11	
Technical Degree	74 (28)	10 (14)	21 (28)	24 (34)	19 (25)	15.5	21.5	
Some Undergraduate Studies	10 (4)	0 (0)	1 (10)	6 (60)	3 (30)	0.5	4.5	
Finished Undergraduate Studies	32 (12)	7 (22)	7 (22)	8 (25)	10 (31)	7	9	
Postgraduate Degree	24 (9)	4 (17)	4 (17)	7 (30)	8 (34)	4	7.5	
Did not disclose	4 (2)	0 (0)	0 (0)	2 (50)	2 (50)	0	2	
Employment situation								0.16
Employed full time	139 (35)	23 (17)	38 (27)	42 (30)	36 (26)	30.5	39	
Employed part-time	9 (42)	3 (33)	4 (45)	2 (22)	0 (0)	3.5	1	
Self-employed	28 (20)	5 (18)	8 (28)	5 (18)	10 (36)	6.5	7.5	
Unemployed	19 (0)	2 (10)	4 (21)	8 (42)	5 (27)	3	6.5	
Retired	32 (0)	11 (34)	13 (41)	5 (15)	3 (9)	12	4	
Regular volunteer	0 (0)	0 (0)	0 (0)	0 (0)	0 (0)	0	0	
Homemaker	33 (0)	13 (40)	4 (12)	7 (21)	9 (27)	8.5	8	
Personal Monthly Income								0.56
Less than $100	58 (22)	16 (28)	10 (17)	14 (24)	18 (31)	13	16	
$101–300	25 (10)	8 (32)	9 (36)	5 (20)	3 (12)	8.5	4	
$301–500	34 (13)	7 (20)	14 (41)	8 (24)	5 (15)	10.5	6.5	
$501–800	80 (30)	14 (18)	21 (26)	26 (32)	19 (24)	17.5	22.5	
$801–1000	28 (11)	2 (7)	8 (29)	6 (21)	12 (43)	5	9	
$1001–2000	12 (5)	3 (25)	4 (33)	3 (25)	2 (17)	3.5	2.5	
$2001–3500	0 (0)	0 (0)	0 (0)	0 (0)	0 (0)	0	0	
$3500+	5 (2)	0 (0)	3 (60)	1 (20)	1 (20)	1.5	1	
Did not disclose	21 (8)	6 (28)	5 (24)	5 (24)	5 (24)	5.5	5	
Family Income Bracket								0.58
Less than $100	14 (5)	6 (43)	4 (29)	1 (7)	3 (22)	5	2	
$101–300	23 (9)	4 (17)	5 (22)	8 (35)	6 (26)	4.5	7	
$301–500	29 (11)	7 (24)	11 (38)	5 (17)	6 (21)	9	5.5	
$501–800	59 (22)	12 (20)	14 (24)	21 (36)	12 (20)	13	16.5	
$801–1000	33 (13)	7 (21)	11 (34)	8 (24)	7 (21)	9	7.5	
$1001–2000	37 (14)	2 (5)	10 (27)	9 (24)	16 (44)	6	12.5	
$2001–3500	14 (5)	3 (21)	4 (28)	4 (29)	3 (22)	3.5	3.5	
$3500+	8 (3)	1 (12)	4 (50)	1 (13)	2 (25)	2.5	1.5	
Did not disclose	46 (17)	22 (48)	6 (13)	10 (22)	8 (17)	14	9	

### Knowledge

**Fig. 1:**
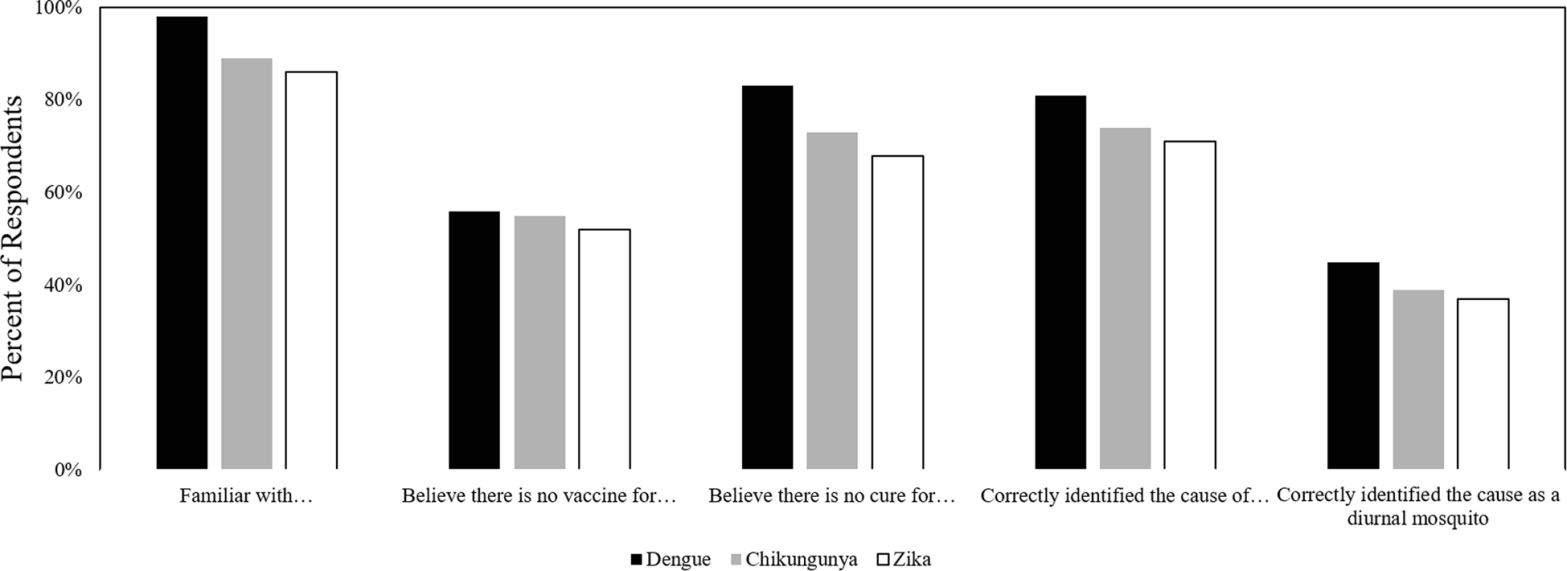
Variation in respondents answers to knowledge questions on DENV, CHIKV, and ZIKV

Knowledge scores were significantly related to the respondent’s age, monthly family income, and marital status (Table 3). Specifically, controlling for other factors, respondents over 70 years of age had knowledge scores lower than respondents in the other age brackets by a factor of 2.14 (Fig. 1; *P <* 0.02). Respondents with a monthly family income of over $2500 had a knowledge score higher than the other income brackets by a factor of 3.53 (*P <* 0.01). Additionally, respondents with a marital status of free union had knowledge scores lower than other marital statuses by a factor of 1.22 (*P <* 0.02). Overall SES group was not associated with log knowledge score, nor was neighborhood, individual monthly income, education level, ethnicity or employment status.

**Table 3:** Significant results of GLM model to predict the knowledge score across all respondents. Other variables were insignificant estimators

Variable	Coefficient	*P*
Over 70 years old	−2.089	0.003
Monthly family income over $2500	3.534	0.011
Marital Status: Free Union	−1.228	0.014
Constant	8.494	0.000

There were more skewed results found within the answers for specific questions (Fig. 1). Overall, 98% of the respondents were familiar with DENV, 89% were familiar with CHIKV, and 86% were familiar with ZIKV (Chi-square: *P* < 0.01). Additionally, 56% of respondents believed there is a vaccine for DENV, 55% believed there is a vaccine for CHIKV, and 52% believed there is a vaccine for ZIKV, though the difference is not statistically significant. Further, 83% believed DENV is curable, 73% believed CHIKV is curable, and 68% believed ZIKV is curable (Chi-square: *P* < 0.01). The majority of respondents correctly identified the primary source of transmission for DENV, CHIKV, and ZIKV with 81, 74, and 71% respectively selecting mosquitoes (Chi-square: *P* < 0.01). For symptoms of the diseases, the average number of correct answers for DENV was 1.9, versus 0.9 for CHIKV and 0.7 for ZIKV. Only 8% of respondents did not select a correct symptom for DENV, compared to 47% for CHIKV and 55% for ZIKV (Chi-square: *P* < 0.01). Lastly, 45% of respondents correctly identified DENV as being transmitted by a diurnal mosquito, compared to 39% for CHIKV and 37% for ZIKV (Chi-square: *P* < 0.01).

### Attitude

Overall, 42% of respondents had attitude scores of 63, meaning they had answered 7 to all questions and were fully worried about contracting any of the diseases, felt that any of the diseases would be a major problem for their health, and were fully likely to seek medical attention if they believed they had contracted any of them. The average value across all respondents was 52. The responses for each specific disease within the first question, how concerned they were about contracting each disease, were highly correlated (> 0.90), indicating minimal variation in responses between diseases for each question. For the question of how much of a major problem contracting each disease would be for their health, responses for each disease were also highly correlated (> 0.83). Correlation among responses for the final question, regarding whether the respondent were likely to seek medical attention, was more varied, with a 0.59 correlation between the DENV responses and both the CHIKV and ZIKV responses despite the CHIKV and ZIKV responses remaining highly correlated (> 0.96). Still there was no significant difference in the mean score between any of the diseases. When asked the number of times they believed they are bitten by a mosquito per day, 54% reported zero, while 33% reported 1–5 times, with the remaining 13% reporting being bit more than five times daily.

**Fig. 2:**
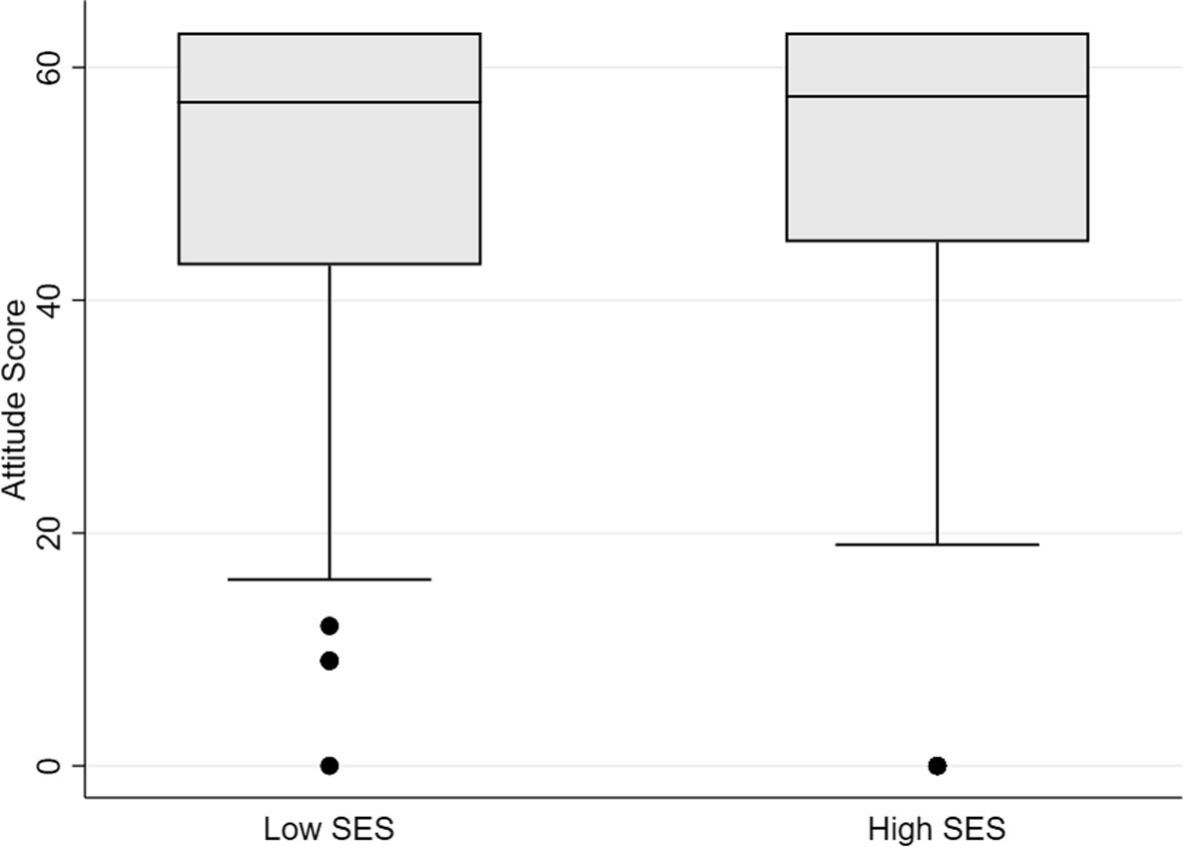
Difference in attitude score across SES groups (*P <* 0.00)

**Fig. 3:**
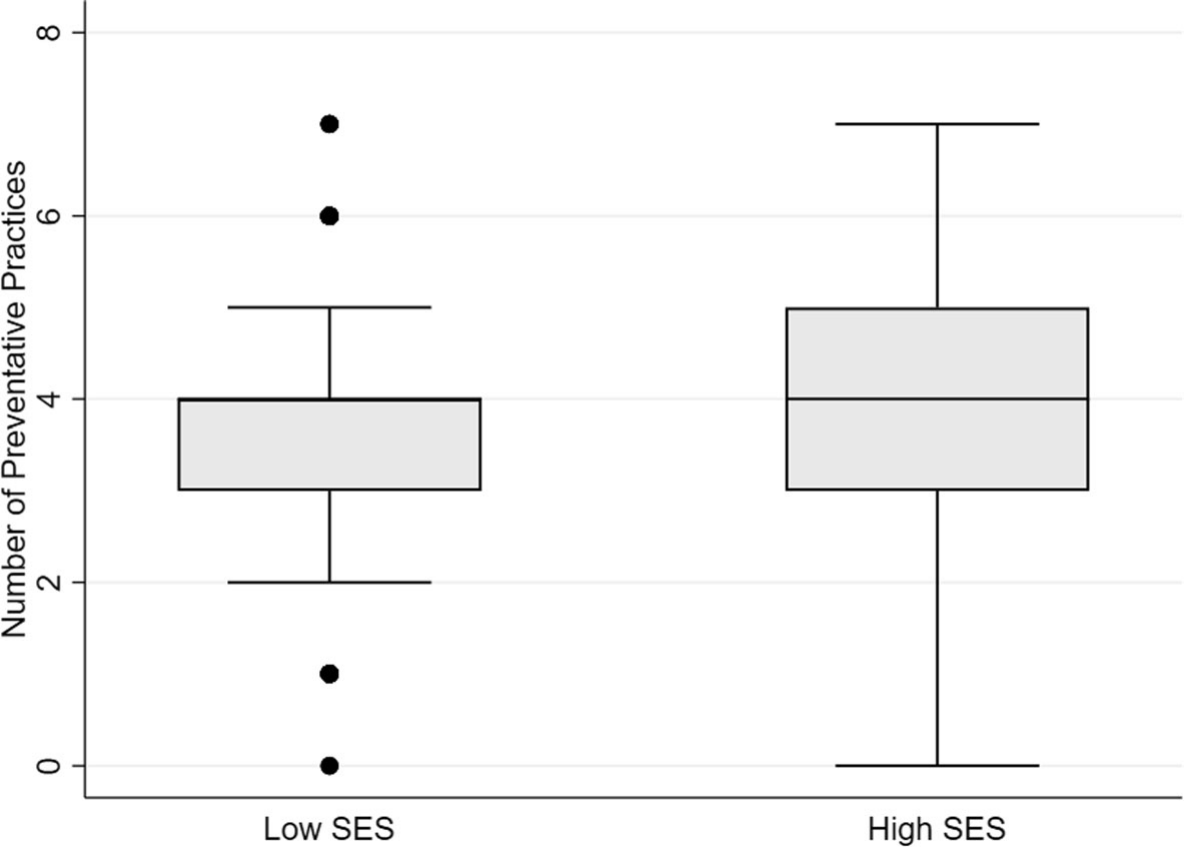
Difference in the number of preventative practices employed by residents in each SES group (*P* < 0.01)

Group SES, monthly family income, and number of times reported being bitten by mosquitoes daily were significant predictors of the attitude score (Table 4). Controlling for all other factors, respondents in the high SES neighborhoods exhibited a significant increase in attitude score (*P* < 0.00) compared to those in the low SES neighborhoods (Fig. 2). Similarly, a monthly family income under $800 led to a significant decrease in the log sum attitude score (*P* < 0.00). Lastly, an increase in the number of times being reported bitten by a mosquito per day led to a significant increase in attitude score (*P* < 0.00). The number of times reported being bitten was also significantly related to neighborhood SES (Fig. 3), as respondents in high SES neighborhoods reported 1.98 daily bites than respondents in low SES neighborhoods (*P* = 0.00).

**Table 4:** Significant results of GLM model to predict the attitude score across all respondents. Other variables were insignificant estimators

Variable	Coefficient	*P*
Number of daily mosquito bites	0.391	0.000
High SES neighborhood	2.909	0.003
Monthly family income under $800	−4.932	0.025
Constant	49.70	0.000

### Practice

A total of 66% of residents reported being aware of measures taken by the local authorities to combat the spread of DENV, CHIKV, and ZIKV. When asked what measures they were aware of, 59% said fumigation, followed by 21% who said either “cleaning” or “clearing trash.” The remaining 20% included respondents being aware of education campaigns, the prevention of water accumulation, and fines. When asked which personal steps they take to avoid contracting the three diseases, an average of 3.7 practices were listed across all respondents. The most commonly reported practice was the elimination of tanks or puddles with stagnant water, selected by 84% of respondents. This was followed by 80% of respondents who reported that they remove containers that may accumulate clean water. Additionally, 61% of respondents reported drinking from cisterns or tanks that are kept tightly closed. Finally, only 50% of respondents reported requesting fumigation services at least once per year and 46% of respondents reported keeping the windows and doors of their homes shut. Sleeping under a bed-net, a practice which would not prevent one from being bitten by the diurnal *Aedes* mosquito, was reported by 21% of respondents. Table 5 indicates the breakdown of each effective preventative practice by focal neighborhood. Overall, respondent’s learned about these measures from an average of 1.5 sources. Television was the most common source of information (56%), followed by medical professionals (26%) and neighborhood campaigns (21%). Less than 10% reported learning about prevention measures from radio, family or friends, work, or other sources respectively. A total of 79% of the respondents believed the practices to be effective and 100% reported that they would seek medical attention if they believed they had contracted one of the three diseases.

**Table 5:** Preventative practices taken by respondents in each focal neighborhood

	Costa Del Este (%)	Punta Pacifica (%)	Altos De Las Torres (%)	Boca La Caja
Frequently change the water in flower vases	2 (5)	7 (12)	17 (40)	7 (47)
Remove containers that accumulate clean water	56 (88)	53 (75)	52 (71)	49 (83)
Eliminate tanks or puddles of stagnant water	56 (88)	57 (81)	56 (76)	49 (83)
Drink from tightly closed water containers	31 (49)	39 (70)	48 (67)	42 (71)
Keep windows/doors closed in the house	35 (55)	29 (41)	30 (45)	22 (39)
Request fumigation	34 (54)	51 (57)	36 (49)	19 (32)

There were no significant predictors of whether the respondent was aware of steps taken by the local authorities to combat the spread of the three diseases. The number of practices taken to avoid contracting the three diseases was significantly related to neighborhood SES and education (Table 6). Controlling for other factors, a respondent in a high SES neighborhood engaged in significantly more practices than a respondent in a low SES neighborhood (Fig. 3; *P* = 0.05). Conversely, respondents whose highest completed education was primary school engaged in significantly fewer practices than those with more education completed (*P* < 0.04). Number of sources of information was significantly related to SES. Controlling for other factors, respondents in a high SES neighborhood received information from 0.49 fewer sources than respondents in low SES neighborhoods (*P* < 0.01). Specific sources of information did not vary significantly between groups nor did the likelihood of finding prevention practices effective. Lastly, respondents whose family earned less than $500 per month were significantly more likely to seek medical attention at a primary health center than respondents who earned over $500 per month (*P* < 0.01). Respondents in the latter group were significantly more likely to seek medical attention at a private hospital than respondents whose family earned less than $500 per month (*P* < 0.01).

**Table 6:** Significant results of GLM model to predict the log number of preventative practices across all respondents. Other variables were insignificant estimators

Variable	Coefficient	*P*
High SES neighborhood	0.320	0.050
Only completed Primary School Education	−1.605	0.050
Constant	4.679	0.000

## Discussion

Our results reveal several key insights regarding the knowledge, attitudes, and practices of residents of four focal neighborhoods of Panama City and the socioeconomic and demographic groups that they belong to. Since knowledge scores were normally distributed and the mean was approximately halfway between selecting none of the correct answers and all of the correct answers, we identify a considerable lack of accurate knowledge regarding the causes, symptoms, and prevention of DENV, CHIKV, and ZIKV. First, over half of the respondents believed that vaccines exist for each disease. While there is a theoretical risk that respondents believed the DENV vaccine available in Brazil at the time was also available in Panama, we find this unlikely given the high percentages of respondents who also thought a CHIKV and ZIKV vaccine existed. Overall, this presents a dichotomy, as this is either an admission that they have not had the supposed vaccine even if though they believe it exists or they are confusing it with other vaccines they may have had. Regardless, this is a considerable piece of misinformation that is pervasive among our sample pool and not restricted to any particular SES or demographic group. This is also an inverse of the more common problem, where the public is unaware of a vaccine that indeed does exist for a particular disease [53–55]. Further qualitative studies with this specific sample are needed to explore beliefs about nonexistent vaccines. Overall, we suggest that future studies carefully word their questions regarding this particular topic so as to avoid confusion on behalf of the respondent and reduce issues in interpretation.

Similarly, at least two-thirds of respondents misleadingly believe that the three diseases are curable, though it is highly possible that respondents confused the idea of “cured” with “treated.” The proportions of respondents who were familiar with each disease, combined with those who correctly identified the lack of vaccine and cure, follows the chronological pattern of the diseases arrive in Panama. DENV, which is endemic [56], commanded the highest awareness and lowest rates of incorrect answers. This was followed by CHIKV [57], which arrived in 2014, and ZIKV which arrived in 2015 [58]. It is expected that respondents would be more knowledgeable about threats that had existed locally for the longest time, though our results indicate that education efforts should not ignore such longstanding threats even amongst the rise of more novel ones. It also raises the key question of when, if ever, does resident KAP regarding a novel threat reach the levels of an endemic threat. Additionally, in light of our results, public education campaigns may be most needed in communities with higher relative proportions of residents over 70 years of age, residents who in free unions, as well as residents whose family earns less than $2500 a month. It is unclear why these specific residents had lower relative knowledge scores, yet both age and income can be key aspects of social determinants of health within a community [59]. Thus, further investigations into these potentially at-risk groups may be warranted.

Our attitude results indicate high levels of concern across our entire sample pool, regardless of specific disease. Respondents were also highly likely to seek out medical attention if they believed they had contracted the disease. Interestingly, the majority of respondents reported being bitten by a mosquito zero times per day on average. While this question is entirely based on the respondent’s perception and is not verified, it does provide an insight into the experience of local residents regarding biting mosquitoes as an aspect of their environment as have other studies on perceptions of vectors and vector-borne disease [60–62]. It is certainly possible that some residents have learned to ignore frequent biting that has simply become a normal component of their lives or that others may be overestimating based on differences in their personal feelings towards biting as a nuisance [63]. Since the response was directly related to neighborhood SES, with residents in the lower SES neighborhoods reporting significantly higher amounts of biting than those in high SES neighborhoods, follow-up studies may seek to investigate this division in perception and whether it is related to actual biting rates. Further, with higher attitude scores related to both high SES neighborhoods and high monthly family income, we illustrate a key difference in the degree of concern between differing groups, as reported biting is higher in low SES neighborhoods but concern is higher in high SES neighborhoods. Addressing heightened concern in high SES neighborhoods should be a focus of local outreach efforts, in addition to education campaigns in low SES neighborhoods that ensure public concern is appropriate.

Practices involving the reduction of standing water were widely reported by all respondents. The elimination of breeding habitat is a key method of reducing local vector abundance [64], and so the high rates of reported utilization of these practices is certainly a positive result for Panama City. However, the relatively low rate of respondents who regularly keep their doors and windows closed is potentially concerning, as mosquitoes often take refuge indoors to escape the heat of the mid-day [65–67]. Follow-up studies would be required to determine whether air conditioning is equally or unequally available across socioeconomic and demographic groups, and if limited access to air-conditioning prompts residents to maintain open doors and windows. An important potential complication to these results could be the type of structure that the respondent resides in. Responsibilities for vector control in some housing structures or communities may fall on building management rather than residents. This is more likely to be the case in the high SES neighborhoods where high rises and gated communities may manage on-site vector control for residents.

With the largest proportion of residents receiving their information from television rather than through other sources, we suggest that outreach and educational messages utilize available broadcast networks in the region. This is in line with other studies, which have also found television to be a key source of information on vector control practice [43, 68, 69]. Since the number of practices taken to avoid contracting the three diseases varied significantly by neighborhood SES, we suggest that greater steps be taken to inform residents in low SES communities of the effectiveness of vector control measures. It is not immediately clear why, when controlling for other factors, individuals with bachelor’s degrees or higher engaged in fewer practices, as other studies have generally found that education is directly related to the participation in such practices [70, 71]. The inverse relationship between respondent’s knowledge score and the number of sources of information indicates that there may be increased risk of misinformation when one diversifies their sources of knowledge on mosquitoes and the viruses they transmit.

## Conclusion

While SES is indeed a cursory label of a community, both attitude and practice scores were predictable at the SES level, while knowledge was only predictable at the individual level.

Our study indicates that low-SES communities with high proportions of low income residents, low education, and elderly residents should be the target of arbovirus prevention programs. In general, these results support our initial hypotheses that lower relative knowledge and fewer practices related to the prevention of DENV, CHIKV, and ZIKV would be found in communities of lower socioeconomic status (SES). We also expected and found relative knowledge about emergent CHIKV and ZIKV to be lower than that of endemic DENV. However, we did not foresee concern to be higher in areas where biting was reported less often, as was found in the high SES neighborhoods. This highlights variation in the experiences of residents in socioeconomically contrasting neighborhoods, and such information must be taken into account when education campaigns are designed. Each community may require programs specifically tailored to meet their needs, based on the particular socioeconomic and demographic proportions of the residents. While the objective of the study was to observe SES-scale or neighborhood-scale effects, we found that there was enough individual-scale diversity across all four neighborhoods for more specific socioeconomic and demographic attributes to be used as determinants of knowledge, attitude, and practice. Thus, we caution future studies seeking to identify generalizations across entire neighborhoods to be cognizant of the level of variation among residents even in seemingly homogenous communities. Overall, with knowledge of CHIKV and ZIKV lower than that of DENV across all of our respondents, we suggest increasing messaging regarding the two more novel threats. Despite CHIKV and ZIKV being present in Panama City for four and 3 years respectively, resident knowledge is still not at the level of DENV. Such information can be helpful in both designing KAP studies as well as educational interventions across Latin America, where CHIKV and ZIKV are emerging threats and differences between socioeconomic groups can be particularly stark. Beneficial follow-up studies and qualitative research would examine a greater variety of neighborhoods, such as some in a more intermediate SES range, as well as investigations into the efficacy of educational campaigns.

## Additional file

Additional file 1: Survey Instrument. (DOCX 74 kb)

## Abbreviations

CHIKV: Chikungunya Virus
DENV: Dengue Virus
HIV/AIDS: Human immunodeficiency virus / Acquired immunodeficiency syndrome
INDICASAT: Instituto de Investigaciones Científicas y Servicios de Alta Tecnología
KAP: Knowledge, Attitude, and Practice
SENACYT: Secretariat for Science, Technology and Innovation of Panama
SES: Socioeconomic status
SNI: Panamanian National System of Investigation
STRI: Smithsonian Tropical Research Institute
ZIKV: Zika Virus
