# Supplementary material for: Socioeconomic and demographic predictors of resident knowledge, attitude, and practice regarding arthropod-borne viruses in Panama
Source: BMC Public Health. 2018 Nov 14;18:1261. doi: 10.1186/s12889-018-6172-4 (PMC6236898; doi:10.1186/s12889-018-6172-4)
Supplement: Supplementary file 1 — Survey Instrument. (DOCX 74 kb) [file 12889_2018_6172_MOESM1_ESM.docx]

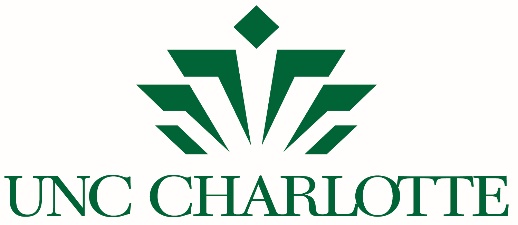


Department of Geography & Earth Sciences

9201 University City Boulevard, Charlotte, NC 28223-0001

t/ 704-678-5973 f/ 704-678-5966 www.geoearth.uncc.edu

# The Socio-Ecology of Mosquito-Borne Diseases

# TO BE COMPLETED BY THE RESEARCH TEAM

# Trap ID_________

**Participant ID** __________

**Community:**

Boca la Caja  Punta Pacifica/Punta Paitilla

Costa del Este  Torrijos Carter/Altos de las Torres

Please take a few minutes to fill out this survey. Your participation is completely anonymous and we will not record your name or contact details. There are no wrong or right answers! Your honest feedback will help us improve health initiatives to protect your community. Thank you for taking part.

## Socio-demographic Information

### Head of household:

Yes  No

### Sex:

Male  Female

### Age:

18-35  36-55  56-70  70+

### Number of people living in the household (including you):

1  2  3

4  5  6

7  more than 7

### Race:

White  African-Caribbean  African-Colonial  Mestizo
 Indigenous  Chinese/Asian  Other: ________________________

### Marital Status:

Single  Married  Divorced

Separated  Widowed  Free union

## Education

### Highest education completed:

None

Some primary school  Finished primary school  Some high-school

Finished high-school  Technical degree  Some UG studies

Finished UG studies  Postgraduate (PG)

## Employment

### Which of the following describes best describes your job situation?

Employed full-time  Employed part-time  Self-employed

Unemployed  Retired  Regular volunteer

Homemaker

### If employed, how many hours a day do you usually work?

0 to 4 hours  4 to 8 hours  more than 8 hours

### How much total income did **you** receive last month, not just from wages or salaries but from all sources – that is, before taxes or other deductions were made?

Less than $100  $101 - $300  $301 - 500

$501 - $800  $801 - $1,000  $1,001 - $2,000

$2,001 – $3,500  above $3,500  Prefer not to say

### How much total income did **your whole family living at home** receive last month, not just from wages or salaries but from all sources – that is, before taxes or other deductions were made?

Less than $100  $101 - $300  $301 - 500

$501 - $800  $801 - $1,000  $1,001 - $2,000

$2,001 – $3,500  above $3,500  Prefer not to say

## Knowledge of Risk

### Which diseases are you familiar with? (check all that apply)

Culebra Fever  Congo River Virus  Zika Virus  Floodwater Fever  Dengue Fever  Chikungunya Virus

### Can these diseases be prevented with a vaccine?

Dengue  Yes  No Do not know

Chikungunya Virus  Yes  No Do not know

Zika Virus  Yes  No Do not know

### Can these diseases be cured?

Dengue  Yes  No Do not know

Chikungunya Virus  Yes  No Do not know

Zika Virus  Yes  No Do not know

How is **Dengue** spread? (**Please select all answers that apply)**

Bite by lice  Dirty water  Mosquito  Bite by ticks

Under-cooked food  Dirty air  Coughing/sneezing

How is **Chikungunya** spread? (**Please select all answers that apply)**

Bite by lice  Dirty water  Mosquito  Bite by ticks

Under-cooked food  Dirty air  Coughing/sneezing

How is **Zika** spread? (**Please select all answers that apply)**

Bite by lice  Dirty water  Mosquito  Bite by ticks

Under-cooked food  Dirty air  Coughing/sneezing

At what time of day are people most likely to be infected by these diseases? (**Please select all answers that apply)**

Dengue  Morning Noon Evening Night

Chikungunya  Morning Noon Evening Night

Zika  Morning Noon Evening Night

## Signs and Symptoms

What are the signs and symptoms of **Dengue**? **(Please select all answers that apply)**

Vomit  Headache Rash  Diarrhea  Chest pain  Muscle pain  Fever  Persistent cough  Conjunctivitis

What are the signs and symptoms of **Chikungunya**? **(Please select all answers that apply)**

Fever  Joint pain Headaches Rash Vomit  Persistent cough

What are the signs and symptoms of **Zika Virus**? **(Please select all answers that apply)**

Chest Pain  Rash  Conjunctivitis  Persistent cough

Headache  Joint pain  Diarrhea  Fever

## Your Attitudes

### How worried are you about contracting these diseases? (1=not worried, 7=extremely worried)

|  | 1 | 2 | 3 | 4 | 5 | 6 | 7 |
| --- | --- | --- | --- | --- | --- | --- | --- |
| DENGUE |  |  |  |  |  |  |  |
| CHIKUNGUNYA |  |  |  |  |  |  |  |
| ZIKA |  |  |  |  |  |  |  |

How much do you believe these diseases are a major problem for your own health? (1=no problem, 7=major problem)

|  | 1 | 2 | 3 | 4 | 5 | 6 | 7 |
| --- | --- | --- | --- | --- | --- | --- | --- |
| DENGUE |  |  |  |  |  |  |  |
| CHIKUNGUNYA |  |  |  |  |  |  |  |
| ZIKA |  |  |  |  |  |  |  |

How likely are you to seek medical attention if you present these diseases symptoms? (1 = not likely, 7 = very likely)

|  | 1 | 2 | 3 | 4 | 5 | 6 | 7 |
| --- | --- | --- | --- | --- | --- | --- | --- |
| DENGUE |  |  |  |  |  |  |  |
| CHIKUNGUNYA |  |  |  |  |  |  |  |
| ZIKA |  |  |  |  |  |  |  |

## Disease Prevention

### Have you or any of your family members had any of these diseases diagnosed/positively tested?

Dengue  Yes  No Do not know

Chikungunya  Yes  No Do not know

Zika  Yes  No Do not know

### If yes, which family member?

You  Husband/wife  Parent  Sibling  Child

### When did they have the disease?

| MONTH | YEAR |
| --- | --- |
|  |  |

### How often does your house/premise get spread with insecticide to kill mosquitoes?

Daily Weekly Monthly Every 6 months Yearly Less than Yearly

### Are you familiar with the measures taken by local authorities to combat these diseases?

Yes  No

### If yes, list which measures:

### ______________________

________________________

________________________

________________________

________________________

### Do you personally take any steps to avoid these diseases?

Yes  No

### If yes, select which measures **(Check all that apply).**

Frequently change the water in flower vases

Sleep under bed nets every night

Remove containers that accumulate clean water (bottles, tires, cans)

Eliminate tanks or puddles with stagnant water

Drink from water containers (cisterns, tanks) tightly closed

Keep windows/doors closed in the house

Request fumigation

Take paracetamol

Others: ____________________________________

### Other: How often do you apply these measures?

|  | **Never** | **Daily** | **Weekly** | **Every 2 weeks** | **Monthly** | **Yearly** |
| --- | --- | --- | --- | --- | --- | --- |
| Frequently change the water in flower vases |  |  |  |  |  |  |
| Sleep under bed nets every night |  |  |  |  |  |  |
| Remove containers that accumulate clean water (bottles, tires, cans) |  |  |  |  |  |  |
| Eliminate tanks or puddles with stagnant water |  |  |  |  |  |  |
| Drink from water containers (cisterns, tanks) tightly closed |  |  |  |  |  |  |
| Keep windows/doors closed in the house |  |  |  |  |  |  |
| Request fumigation |  |  |  |  |  |  |
| Take paracetamol |  |  |  |  |  |  |
| Other: _______________________________________ |  |  |  |  |  |  |

### How did you learn about these measures? **(Check all that apply)**

TV  Radio  Newspaper

At work  In school  Neighborhood campaign

From a friend/family member  From a medical professional  Other, specify:_________________

### Do you think any of these measures are useful at limiting these diseases?

Yes  No

### If no, provide a reason:

_________________________________________________________________________________________________

### If you or any of your family felt sick and thought it was one of these diseases, would you go for treatment?

Yes  No

### If yes, where would you go?

Centro de Salud  Private Hospital  Public Hospital (e.g. Santo Tomás)

Caja del Seguro Social  Other, specify: _____________

### In any given day, how many times do you think you get bitten by mosquitoes between 6am and 4pm (morning and afternoon)? Please write an average number of bites per day: _________________

### If no, list reasons why:

________________________

________________________

________________________

**Thank you for taking the time to fill out our survey.**

**Your input is greatly appreciated.**
